# Role of minimally invasive surgery versus open approach in patients with early-stage uterine carcinosarcomas: a retrospective multicentric study

**DOI:** 10.1007/s00432-020-03372-x

**Published:** 2020-09-03

**Authors:** Giacomo Corrado, Francesca Ciccarone, Francesco Cosentino, Francesco Legge, Andrea Rosati, Martina Arcieri, Luigi Carlo Turco, Camilla Certelli, Alex Federico, Enrico Vizza, Francesco Fanfani, Giovanni Scambia, Gabriella Ferrandina

**Affiliations:** 1 Department of Woman, Child Health and Public Health, Gynecologic Oncology Unit, Fondazione Policlinico Universitario A. Gemelli, IRCCS, Rome, Italy; 2 Department of Gynecologic Oncology, Gemelli Molise Spa, Università Cattolica del Sacro Cuore, Campobasso, Italy; 3 Gynecologic Oncology Unit, “F. Miulli” General Regional Hospital, Acquaviva Delle Fonti, Bari, Italy; 4 Department Gynecology and Breast Care Unit, Mater Olbia Spa, Olbia, Italy; 5 Department of Experimental Clinical Oncology, Gynecologic Oncology Unit, “Regina Elena” National Cancer Institute, IRCCS, Rome, Italy; 6 Istituto di Ostetricia e Ginecologia, Fondazione Policlinico Universitario A. Gemelli, IRCCS, Università Cattolica del Sacro Cuore, Rome, Italy

**Keywords:** Uterine carcinosarcoma, Minimally invasive surgery, Open surgery

## Abstract

**Objective:**

The aim of this retrospective study was to compare surgical and survival outcome in only patients with early-stage UCSs managed by laparotomic surgery (LPT) versus minimally invasive surgery (MIS).

**Methods:**

Data were retrospectively collected in four Italian different institutions. Inclusion criteria were UCS diagnosis confirmed by the definitive histological examination, and stage I or II according to the FIGO staging system.

**Results:**

Between August 2000 and March 2019, the data relative to 170 patients bearing UCSs were collected: of these, 95 were defined as early-stage disease (stage I–II) based on the histological report at the primary surgery, and thus were included in this study. Forty-four patients were managed by LPT, and 51 patients were managed by MIS. The operative time was lower in the MIS group versus the LPT group (*p* value 0.021); the median estimated blood loss was less in the MIS group compared to the median of LPT group (*p* value < 0.0001). The length of hospital stay days was shorter in the MIS patients (*p* value < 0.0001). Overall, there were eight (8.4%) post-operative complications; of these, seven were recorded in the LPT group versus one in the MIS group (*p* value 0.023). There was no difference in the disease-free survival (DFS) and overall survival (OS) between the two groups.

**Conclusion:**

There was no difference of oncologic outcome between the two approaches, in face of a more favourable peri-operative and post-operative profile in the MIS group.

## Introduction

Uterine carcinosarcomas (UCSs) are rare and aggressive malignancies characterized by the concomitant presence of carcinomatous and sarcomatous components (Akahira et al. 2006; Cantrell et al. 2015). UCSs account for less than 5% of all uterine tumors, however, a recent study conducted in the USA has reported that the rate of this disease has significantly increased over time, possibly due to the increase of number of older patients, obesity, increased use of tamoxifen, etc. (Pothuri et al. 2006; Onstad et al. 2016; Matsuo et al. 2018). Even though UCSs are considered as metaplastic endometrial carcinomas (EC), their aggressiveness is much higher compared to the classical EC; indeed, the proportion of stage I–II is lower in UCSs compared to EC, and, even in early stage, UCSs patients experience worse prognosis with a 5-years survival of 60% (Gonzalez Bosquet et al. 2010).

In addition, high grade, older age, and lymphovascular space invasion are more frequently documented in UCSs than in other EC types (Cantrell et al. 2015; Abdulfatah et al. 2019). In this context, it has also to be acknowledged that UCSs display a mutational profile endowed with high copy number, and unfavourable clinical outcome (The Cancer Genome Atlas Research Network and Levine 2013; Leskela et al. 2019; Carlson and McCluggage 2019).

In early-stage disease, surgery represents the milestone of treatment, and includes total hysterectomy, bilateral salpingo-oophorectomy, pelvic and aortic lymphadenectomy, and peritoneal biopsies (Baekelandt and Castiglione 2009; Denschlag and Ulrich 2018) www.nccnguidelines.gov (2020); even though omentectomy or omental al sampling are not formally recommended, these procedures often carried out probably because microscopic involvement has been shown to account for 35% of omental disease, and thus could be missed (Ross et al. 2018).

The most frequent surgical approach adopted in early UCSs is laparotomy, and few retrospective data are available relative to the minimally invasive surgery in UCSs (Wallwiener et al. 2016; Tan et al. 2017; Dellinger et al. 2017); within the GOG LAP2 trial (Walker et al. 2009), which has investigated the clinical outcome of early-stage EC patients triaged to laparoscopic versus laparotomic surgery, the sub-group analysis of “high risk” histotypes (i.e. clear cells, serous, and UCSs) reported similar rates of recurrence and survival between the two approaches (Fader et al. 2016).

The aim of this retrospective study was to compare surgical and survival outcome in only patients with early-stage UCSs managed by laparotomic surgery (LPT) versus minimally invasive surgery (MIS).

## Patients and methods

Data were retrospectively collected in four different institutions: “Fondazione Policlinico Universitario A. Gemelli” Rome, “Regina Elena National Cancer Institute” Rome, “Gemelli Molise spa, Università Cattolica del Sacro Cuore” Campobasso, and the “Miulli hospital”, Acquaviva delle Fonti in Bari. This study was approved by the internal review board at each participating institution. All patients had already provided a written informed consent for their data to be collected and analysed for scientific purpose, according to our institutional policy.

Inclusion criteria were UCS diagnosis confirmed by the definitive histological examination, and stage I or II according to the FIGO staging system (Creasman 2009).

Patients with anesthesiological contraindications for the MIS approach (i.e. patients who could not sustain a steep Trendeleburg position) were triaged to laparotomic surgery. Previous abdominal surgery was not considered an exclusion criterion.

All patients were evaluated before surgery by means of medical history, physical examination, vaginal–pelvic examination, chest X-ray or chest CT, pelvic ultrasound scans and complete abdomen and pelvis CT scan or MRI; PET/CT scans if any suspicious images in previous examinations.

Details relative to the type of hysterectomy according to the Querleu-Morrow classification (Querleu and Morrow 2008), and lymph node assessment (i.e. systematic lymphadenectomy or lymph node sampling or sentinel lymph node technique (SLN) were collected in both groups.

Intraoperative and postoperative complications were defined according to Common Terminology Criteria for Adverse Events (CTCAE) version 4.3 (U.S. Department of Health and Human Services 2009).

Adjuvant therapy was tailored to the pathologic findings at the primary surgery after multidisciplinary tumor board (gynecologic oncology, pathology, radiation oncology, medical oncology) discussion. Adjuvant chemotherapy including always platinum/paclitaxel or just platinum in unfit patients. The vast majority of adjuvant radiotherapy was represented by external beam pelvic irradiation (45–50 Gy) plus brachytherapy in case of cervical involvement. Treatment was based on the National Comprehensive Cancer Network (NCCN) guidelines (www.nccn.org.*professionals.physician_gls*) as well as ESGO, and ESTRO guidelines (Baekelandt and Castiglione 2009; Creasman 2009; Plataniotis and Castiglione 2010; Colombo et al. 2016).

The data relative to the time interval from surgery to the start of adjuvant therapy were calculated, if available. Follow-up data were recorded through phone calls, if not available from medical records.

Follow-up schedule include clinical and gynecological examination, CA125 level and ultrasound pelvic scan every 3 months in the first 2 years and every 6 months thereafter; abdomen and thorax CT scan after the end of adjuvant treatment and every 6 months in the first 2 years and every 12 months thereafter. In case of undefined lesions to ultrasound or CT scan, it is scheduled a PET/CT.

### Statistical analysis

Descriptive analysis of data was carried out by Fisher’s exact test for proportion for categorical data or Wilcoxon rank sum non-parametric test for continuous variables. Disease-free survival (DFS) would be calculated from the date of surgery up to disease progression or the date last seen; overall survival (OS) would be calculated from the date of surgery up to death of disease or the date last seen. Survival estimates would be analysed by the Kaplan–Meier method, and the log rank test would be used to assess statistical significance. Multivariate analysis of prognostic factors would be carried out by the Cox regression model. The SPSS software (SPSS version 21.0, SPSS Inc., Chicago, Illinois, USA) was used for all statistical evaluations.

## Results

Between August 2000 and March 2019, the data relative to 170 patients bearing UCSs were collected; of these, 95 were defined as early-stage disease (stage I–II) based on the histological report at the primary surgery, and thus were included in this study. Forty-four patients were managed by LPT, and 51 patients were managed by MIS. About 75% of patients have been enrolled in the last 10 years.

Table 1 shows the patients features in the whole series, as well as in the LPT and MIS groups: in the whole series, the median age was 67 years (range 39–88), the median BMI was 28 kg/m^2^ (range 19–47), and the vast majority of patients (*N* = 73, 76.8%) referred comorbidities; age, BMI, and proportion of patients with comorbidities did not differ between the LPT group and the MIS one.

**Table 1:** Patient characteristics

	*N*	LPT *N*. (%)	MIS *N*. (%)	*p* value^a^
All	95	44	51	
Age, years				
Median (range)	67 (39–88)	66 (50–88)	68 (39–88)	0.51
BMI, kg/m^2^				
Median (range)	28 (19–47)	28 (19–45)	28 (22–47)	0.67
Patients with morbidity				
No	22	12 (27.3%)	10 (19.6%)	0.28
Yes	73	32 (72.7%)	41 (80.4%)	
Type of hysterectomy^b^				**0.042**
A	81	34 (77.3%)	47 (92.1%)	
B1	14	10 (22.7%)	4 (7.8%)	
Pelvic lymph node assessment				0.83^c^
No	39	19 (43.2%)	20 (39.2%)	
Sampling	9	4 (9.1%)	5 (9.8%)	
Lymphadenectomy	47	21 (47.7%)	26 (51.0%)	
No. of pelvic lymph nodes removed				**0.016**
Median (range)	16 (2–59)	21 (2–59)	12 (2–32)	
Aortic lymph node assessment				0.37^c^
No	82	36 (81.8%)	46 (90.2%)	
Sampling	10	7 (15.9%)	3 (5.9%)	
Lymphadenectomy	3	1 (2.3%)	2 (3.9%)	
No. of aortic lymph nodes removed				0.33
Median (range)	6 (3–27)	8.5 (5–27)	6 (5–16)	
Omentectomy				**0.044**
No	88	38 (86.4%)	50 ()	
Yes	7	6 (13.6%)	1 ()	
Stage				0.51
IA	43	21 (47.7%)	22 (43.1%)	
IB	32	16 (36.4%)	16 (31.4%)	
II	20	7 (15.9%)	13 (25.5%)	
Tumor diameter, cm				** < 0.0001**
Median (range)	5.7 (1–20)	7 (1–20)	5 (2–12.5)	
LVSI				0.51
No	56	28 (63.6%)	28 (54.9%)	
Yes	39	17 (36.4%)	22 (45.1%)	
Adjuvant treatment				0.8
No	21	9 (20.4%)	12 (27.3%)	
Yes	74	35 (79.5%)	39 (72.7%)	
Radiotherapy	15	6	9	
Chemotherapy	29	17	12	
Chemotherapy + radiotherapy	30	12	18	

^a^calculated by the χ2 test or the Fisher’s exact test for proportions, and the Wilcoxon rank sum test for continuous data; ^b^according to the Querleu-Morrow classification^21^; ^c^calculated based on no lymph node assessment versus lymph node assessment

LPT: laparotomic surgery, MIS: Minimally invasive surgery; BMI: Body Mass Index

As far as surgical procedures are concerned, the vast majority of patients in the whole series (*N* = 81, 85.3%) underwent type A hysterectomy; type B1 hysterectomy was less frequently carried out in the MIS group than in the LPT group (*p* value = 0.042). In the whole series, assessment of pelvic lymph node status was carried out by lymphadenectomy (*N* = 47, 49.5%), or sampling (*N* = 9, 9.5%), while very old and/or unfitted patients, and patients intraoperatively judged to harbour excessive tissue fragility (*N* = 39) were not triaged to pelvic lymph node assessment; there was no difference in the distribution of lymph node procedures between the LPT and the MIS group (*p* value 0.83). Conversely, the number of pelvic lymph nodes removed was lower in the MIS group (median = 12, range 2–32) compared to the LPT one (median = 21, range 2–59) compared (*p* value 0.016).

Aortic lymph node status assessment was carried out in only 13 patients, due to suspicious lymph nodes at surgery (LPT 8, MIS 5). In the whole series, the median number of aortic lymph nodes removed was six (range 3–27) and did not differ between the two groups. Omentectomy was carried out in only seven patients (7.4%).

Pathologically assessed stage was: stage IA (*N* = 43), IB (*N* = 32), II (*N* = 20); stage II was more frequent in the MIS group compared to the LPT group, but the *p* value was not significant (*p* value = 0.51). All pelvic and aortic lymph nodes were histologically negative.

The tumor diameter was demonstrated to be larger in the LPT patients compared to the MIS group (median size 7 cm, range 1–20 versus median size 5 cm, range 2–12.5, *p* value < 0.0001). The rate of positive lymphovascular space invasion (LVSI) was shown in 41.5% of all patients and did not differ between the two groups.

### Adjuvant treatment

As shown in Table 1, in the whole series, 21 patients were triaged to surveillance due to very old age, and/or unsatisfactory performance status (*N* = 19), or refusal (*N* = 2), while 73 patients were administered adjuvant treatment; there was no difference in the distribution of adjuvant treatment between the two groups. Of patients undergoing adjuvant treatment, 15 (20.3%) were administered radiotherapy, 29 (39.2%) were treated by only chemotherapy, and 30 (40.5%) were treated by chemotherapy followed by radiotherapy. There was no difference in the time interval from completion of surgery and start of adjuvant treatment between the two groups; the median time from surgery to the start of the adjuvant therapy was 6 weeks in both groups (data not shown).

### Surgical details and intraoperative and postoperative morbidity

Surgical details are shown in Table 2: the operative time (OT) was lower in the MIS group versus the LPT group (median = 130 min, range 50–400 versus media*N* = 180 min, range 60–300) (*p* value = 0.021); the median estimated blood loss (EBL) was 50 cc (range 10–600) in the MIS group compared to the median of 100 cc (range 100–2500) in the LPT group (*p* value < 0.0001) (Table 3).

**Table 2:** Surgical details, peri-operative and post-operative complications

	All (*N* = 95)	LPT (*N* = 44)	MIS (*N* = 52)	*p* value^a^
Operative time, min				**0.021**
Median (range)	150 (50–400)	180 (60–300)	130 (50–400)	
Estimated blood loss, cc				** < 0.0001**
Median (range)	100 (10–2500)	200 (100–2500)	50 (10–600)	
Length of hospitalization, days				** < 0.0001**
Median (range)	4 (2–21)	6.5 (2–21)	3 (2–10)	
Patients with intra-operative complications	1	1	-	
Right obturator vein lesion	1	1	-	
Patients with post-operative complications	8	7	1	**0.023**^b^
Grade II				
Atrial fibrillation	1	1	-	
Lung failure and delirium	1	1	-	
Lower limb neuropathy	1	–	1	
Wound dehiscence	1	1	-	
Grade III				
Pelvic lymphocele requiring drainage	1	1	–	
Hemoperitoneum	1	1	–	
Grade V				
Death (duodenal perforation)	1	1	–	
Death (abdominal abscess)	1	1	–	

^a^calculated by the Wilcoxon rank sum test for continuous data; ^b^calculated by the Fisher’s exact test

As far as the hospital stay is concerned, the number of days was shorter in the MIS patients (median = 3, range 2–10) versus the LPT group (median = 6.5, range 2–21) (*p* value < 0.0001). Only 1 intra-operative complication was documented: it occurred in the LPT group, and consisted in the lesion of the right obturator vein which was promptly repaired, though resulting in 2500 cc EBL. Overall, there were (15.0%) post-operative complications; they were more frequent in the LPT group (*N* = 7, 15.9%) versus the MIS group (*N* = 1, 2.0%) (*p* value = 0.023) (Table 2). In particular, in the LPT group there were five surgical complications (grade 2 wound dehiscence, grade lymphocele, grade 3 hemoperitoneum, grade 5 duodenal perforation leading to death, and grade 5 abdominal abscess with death); there were also three medical complications including grade 2 atrial fibrillation, and grade 2 lung failure in the LPT group, and only one grade 2 lower limbs neuropathy in the MIS group.

### Clinical outcome

As for April 2020, follow-up was available in 91 patients due to the loss of data for 4 patients; overall, the median follow-up was 18 months (range 1–212), but patients managed by MIS were operated in more recent years compared to the LPT patients (i.e. the percentage of MIS patients operated since 2010 was 93.7% versus 55.0% in the LPT group).

Overall, the number of recurrences was 34 (37.4%). Of these, 16/43 (37.2%) were documented in the LPT group, and 18/48 (37.5%) were in the MIS group. Overall, the most frequent site of recurrence was the central pelvic area (*N* = 13) followed by distant sites (*N* = 12), and lymph nodes (*N* = 8) (Table 3).

**Table 3:** Pattern of recurrences

	All patients *N* = 91	LPT *N* = 43 (%)	MIS *N* = 48 (%)	*p* value
Recurrence				
No	53 ()	27 (62.8%)	30 (62.5%)	0.76
Yes	34 ()	16 (37.2%)	18 (37.5%)	
Central pelvic	11	6	7	
Lymph node	8	4	4	
Distant disease	9	5	7	
Unknown	1	1	–	

**Fig. 1:**
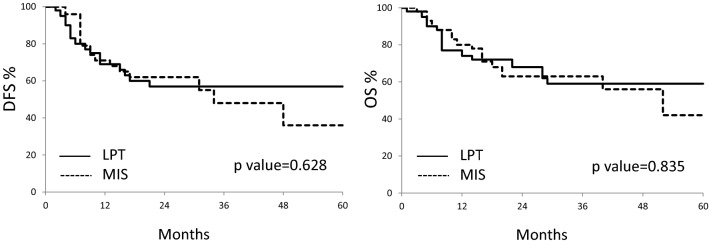
Kaplan–Meier curves relative to DFS (**a**), and OS (**b**) according to the surgical approach

As shown in Fig. 1a, there was no difference in the disease-free survival (DFS) between the two groups; the 2-years DFS rate was 57% in the LPT group versus 62% in the MIS group (*p* value 0.628). Death of disease was registered in 31 patients (34.1%); the 3-years OS rate was 59% in the LPT group versus 63% in MIS groups (*p* value = 0.835) (Fig. 1b).

Univariate analysis of variables conditioning DFS in the whole series showed that only stage II was associated with statistically significant worse prognosis (*p* value 0.0002) (Table 4); in the multivariate analysis, stage II and tumor size were shown to play an independent, unfavorable role (Table 4). Univariate analysis of variables conditioning OS in the whole series showed that only stage II was associated with statistically significant worse prognosis; in the multivariate analysis, stage II and tumor size were associated with unfavorable clinical outcome (data not shown).

**Table 4:** Univariate and multivariate analysis of features conditioning PFS in the whole series (*N* = 80)

Variable	Univariate			Multivariate		
	*β*	Hazard ratio (CI 95%)	*p *value^a^	*β*	Hazard ratio (CI 5%)	*p *value^a^
Age						
(≤ 67 years vs. > 67 years)	0.349	1.418 (0.719–2.796)	0.313	0.242	0.875 (0.324–1.902)	0.592
Stage						
(I vs. II)	1.100	3.003 (1.495–2.796)	**0.002**	1.551	4.715 (1.837–12.099)	**0.001**
Size of tumor						
(≤ 60 mm vs. > 60 mm)	0.567	1.762 (0.847–3.666)	0.129	1.342	3.826 (1.457–10.047)	**0.006**
LVSI						
** (**No vs. yes)	− 0.116	0.890 (0.437–1.812)	0.748	− 0.817	0.442 (0.186–1.046)	0.079
Surgical approach						
(LPT vs. MIS)	0.166	1.180 (0.599–2.326)	0.632	0.810	2.240 (0.850–5.947)	0.102

^*a*^calculated by Cox regression

*LVSI* lymphovascular space involvement, *MIS* minimally invasive surgery, *LPT* laparotomy

## Discussion

UCSs are aggressive tumors, and are included in the “high-risk” group according to the ESMO-ESGO-ESTRO classification (Colombo et al. 2016); however, the clinical outcome of UCSs is even worse than clear cell, and serous EC (Koskas et al. 2016; Fader et al. 2016), thus emphasizing the need to better define the role of MIS focusing on this specific clinical setting. The current NCCN guidelines relative to all “high risk” EC suggest that MIS should be the preferred approach when technically feasible, based on a few retrospective studies (Fader et al. 2012; Koskas et al. 2016; Monterossi et al. 2017; Salehi et al. 2017), and on the sub-analysis of the prospective phase III LAP2 trial (Fader et al. 2016). Also, the LACE trial shows as in the endometrial cancer stage I MIS improves the quality of life after surgery, decreases risk of major surgical adverse events without impacting on disease-free survival at 4.5 years and on overall survival (Janda et al. 2010, 2017; Obermair et al. 2012).

However, the studies specifically focused on the role of MIS versus LPT only in UCSs are few, and also include advanced stage of disease (Fader et al. 2012; Koskas et al. 2016; Salehi et al. 2017).

To our knowledge, our study represents the largest, retrospective series comparing the role of MIS versus LPT only in pathologically assessed stage I–II disease UCSs: there was no difference in terms of oncologic outcome between the two approaches, in face of a more favourable peri-operative and post-operative profile in the MIS group.

As far as the surgical procedures are concerned, the rate of type B1 hysterectomy was lower in the MIS patients, a finding which could be ascribed to the smaller tumor diameter in the MIS group, as also suggested in previous studies (Koskas et al. 2016). Assessment of pelvic lymph node status was carried out in about 60% of overall patients; in this context, we have to acknowledge that > 75% of our patients had comorbidities, and around one-third were obese, and this could have led to spare patients pelvic lymphadenectomy to limit the operative time, and also reduce surgical morbidity since the high aggressiveness of this histological subtype strongly increases the probability that patients would require adjuvant treatment. Finally, it has been recognized that in the real-world practice, only 35–57% of patients in some gynecologic oncology centres in USA were triaged to lymphadenectomy, as summarized in the Vorgias review (Vorgias and Fotiou 2010); as a matter of fact, even considering the large series from the SEER database (*N* = 1885 patients), and the Netherland Cancer registry (*N* = 1140 patients), the “lymphadenectomy issue” still remains controversial (Nemani et al. 2008; Versluis et al. 2018); indeed, the SEER study concluded that lymphadenectomy is associated with improved overall survival with no benefit associated to adjuvant radiotherapy (Nemani et al. 2008), while the Dutch study demonstrates that (1) lymphadenectomy is related to improved survival only if > 10 lymph nodes are removed, and (2) adjuvant therapy improves survival when lymphadenectomy is omitted, or when lymph nodes are positive (Versluis et al. 2018). Probably, these conflicting findings could be related to the fact that the two studies included also stage III (Nemani et al. 2008; Versluis et al. 2018), and even stage IV disease (Versluis et al. 2018). Indeed, in the large series including 5614 stage I UCS patients, lymphadenectomy ≥ 15–20 lymph nodes removed was associated with better survival (Seagle et al. 2017).

Some recent lines of evidence have been reported relative to the possibility to adopt the sentinel lymph node mapping (SLN) also in UCS patients (Schiavone et al. 2016), as also recommended for low-risk EC (Leitao et al. 2013; Holloway et al. 2017); Indeed, Schiavone et al. (2016) reported similar progression-free survival in UCSC patients (stage I–IV) undergoing SLN and adjuvant therapy compared to systematic lymphadenectomy. The confirmation of these findings in a larger series could lead to guarantee the staging of disease while sparing potential morbidities.

As expected, MIS provided reduced median operative time, and blood loss, and also guaranteed a faster return home, as largely reported (Walker et al. 2009; Fader et al. 2016).

There was only one intraoperative complication in the LPT group; on the other hand, seven (15.9%) postoperative complications were registered in the LPT group versus only one postoperative complications in the MIS one (*p* value 0.023): of these, five were surgical morbidities including three patients with grade 3 morbidities, and two patients experiencing grade 5 morbidities leading to death.

The short hospitalization interval, and the low postoperative morbidity profile may fasten the administration of adjuvant treatment, which was carried out in approximately 75% of our patients; the distribution of treatments was similar between the two groups, as well as the time interval from surgery to the start of the adjuvant therapy. However, due to the retrospective design of our study, some data about adjuvant therapy were missed, thus representing a bias of the study. In particular, it has been impossible to find accurate radiotherapy data.

As far as the oncologic outcome is concerned, we have to acknowledge that the patients managed by MIS were operated in a more recent time frame, thus leading to shorter follow duration; overall, relapse of disease occurred in was 37.4%, a figure which appears higher compared to the literature; however, the percentage of stage II was higher than expected.

In our series, multivariate analysis led to define stage II of disease, and tumor size as independent, unfavourable prognostic parameters for DFS and OS; this could be ascribed to the fact that this study has focused only on pathologically assessed stage I–II patients, in which the lymph node status and extrauterine disease was not contemplated. Despite the bias potentially associated with the retrospective design and the sample size, we demonstrated that the MIS approach was shown not to be detrimental to the clinical outcome.
